# Dyslipidemia, chronic inflammation, and subclinical atherosclerosis in children and adolescents infected with HIV: The PositHIVe Health Study

**DOI:** 10.1371/journal.pone.0190785

**Published:** 2018-01-10

**Authors:** Luiz Rodrigo Augustemak de Lima, Edio Luiz Petroski, Yara Maria Franco Moreno, Diego Augusto Santos Silva, Erasmo Benício de Moraes Santos Trindade, Aroldo Prohmann de Carvalho, Isabela de Carlos Back

**Affiliations:** 1 Research Centre for Kinanthropometry and Human Performance. Department of Physical Education. Federal University of Santa Catarina. Florianópolis, Santa Catarina, Brazil; 2 Department of Nutrition and Postgraduate Program in Nutrition. Federal University of Santa Catarina. Florianópolis, Santa Catarina, Brazil; 3 Department of Pediatrics, Medical School. Federal University of Santa Catarina. Florianópolis, Santa Catarina, Brazil; 4 Hospital Infantil Joana de Gusmão. Florianópolis, Santa Catarina, Brazil; Azienda Ospedaliera Universitaria di Perugia, ITALY

## Abstract

HIV-infected children and adolescents may be at risk for cardiovascular disease due to chronic inflammation and exacerbation of risk factors. The aim of this study was as follows: 1) compare cardiovascular risk factors, chronic inflammation, and carotid intima-media thickness (IMTc) between the HIV and control groups; 2) determine the association of HIV and antiretroviral (ART) regimens with cardiovascular risk factors, chronic inflammation, and IMTc; and 3) identify variables associated with elevated IMTc. Cross-sectional analysis of 130 children and adolescents, 8–15 years of age, divided into HIV-infected (n = 65) and healthy control (n = 65) participants. Body fat, blood pressure, glycemia, insulin, and glycated hemoglobin, total cholesterol and fractions (LDL-C and HDL-C), triglycerides, C-reactive protein (CRP), interleukin (IL)-6, tumor necrosis factor-alpha (TNF-α), and the IMTc were measured. The results showed HIV-infected children and adolescents had higher levels of glycemia (87.9 vs. 75.9 mg.dL^−1^, p< 0.001), LDL-c (94.7 vs. 79.5 mg.dL^−1^, p = 0.010), triglycerides (101.2 vs. 61.6 mg.dL^−1^, p< 0.001), CRP (1.6 vs. 1.0 mg.L^−1^, p = 0.007), IL-6 (1.42 vs. 0.01 pg.mL^−1^, p< 0.001), TNF-α (0.49 vs. 0.01 pg.mL^−1^, p< 0.001), mean IMTc (0.526 vs. 0.499 mm, p = 0.009), and lower HDL-c (53.7 vs. 69.4 mg.dL^−1^, p< 0.001) compared to controls. Systolic blood pressure (β = 0.006, p = 0.004) and TNF-α (β = −0.033, p = 0.029) accounted for 16% of IMTc variability in HIV-infected children and adolescents. In patients using protease inhibitors-based ART, male gender (β = −0.186, p = 0.008), trunk body fat (β = −0.011, p = 0.006), glucose (β = 0.005, p = 0.046), and IL-6 (β = 0.017, p = 0.039) accounted for 28% of IMTc variability. HIV-infected children and adolescents may be at risk for premature atherosclerosis due to chronic inflammation and dyslipidemia. Interventions with the potential to improve lipid profile, mitigate inflammation, and reduce cardiovascular risk are needed.

## Introduction

Children and adolescents infected with HIV by mother-to-child transmission are exposed to the adverse effects of HIV, related complications, and adverse reactions of treatment since conception. Among the factors that may be implicated in atherosclerotic disease, dyslipidemia and inflammation are the most important [1–5]. The abnormalities in carbohydrate metabolism are evident [2–5], but less common complications [6], although other studies have not demonstrated any impairment [7, 8]. The occurrence of these cardiovascular risk factors in childhood and adolescence has been associated with intima-media thickening of the carotid artery (IMTc) [9].

Since atherosclerosis is largely inflammatory, research involving HIV-infected adults has shown the potential predictor of mortality of chronic inflammation, regardless of traditional cardiovascular risk factors [10]. However, there is a lack of consensus whether HIV-infected children and adolescents have increased levels of C-reactive protein (CRP) [2, 11–17], interleukin (IL)-6 [11, 16, 17], and tumor necrosis factor-alpha (TNF-α) [11, 18] when compared to healthy controls. Therefore, there is a need for studies to test the association between HIV and inflammation biomarkers, with a control group as a reference, also verifying the contribution of cardiovascular risk factors and inflammation to IMTc.

HIV-infected children and adolescents have been shown to have elevated IMTc in some studies [12–15], even in patients with an undetectable viral load when compared to the controls [15]. Furthermore, in the detailed analysis, nadir CD4 lymphocytes and the duration of protease inhibitors (PI) on antiretroviral therapy (ART) were also associated with IMTc [15]. In the 2000s our group found an association between increased IMTc and a higher CD8 lymphocyte count, suprailiac skinfold, cardiac mass index, respiratory frequency, stavudine use, lower CD4 T-cell count, and total cholesterol [13]. The current availability of new drugs and ART regimens highlights the importance to investigate the behavior of associations of cardiovascular risk factors and clinical variables with subclinical atherosclerosis in pediatric patients. Moreover, identifying the factors that contribute to the development of atherosclerotic diseases in HIV-infected children and adolescents represents the opportunity to prevent and ultimately prolong life.

Considering the inconsistency of the literature on cardiovascular risk factors and inflammation, the aim of this study was to determine the following: 1) compare cardiovascular risk factors, chronic inflammation, and IMTc among HIV-infected children and adolescents with the control group; 2) compare the presence and severity of cardiovascular risk factors, biomarkers of inflammation, and IMTc in different ART regimens; and 3) identify the variables associated with the increase in the IMTc in HIV-infected children and adolescents.

## Materials and methods

### Study design

The PositHIVe Health Study was a cross-sectional study with a focus on lifestyle, physical fitness, and cardiovascular risk factors in HIV-infected children and adolescents (HIV group) and healthy controls (control group). The study was conducted in Florianópolis, Brazil from November 2015 to September 2016. The city has a Human Development Index (HDI) of 0.847. The participants in both groups resided in Florianópolis and the metropolitan region (HDI = 0.739–0.809). These values are high, above of average in Brazil (HDI = 0.727), and they represent lower infant mortality and longevity, more education, and income. The university’s Institutional Review Board and the Hospital Ethics Committee approved the study (No. 49691815.0.0000.0121). Written informed consent was obtained from the parent(s) or legal guardian(s) of the participants.

### Participants

**Fig 1:**
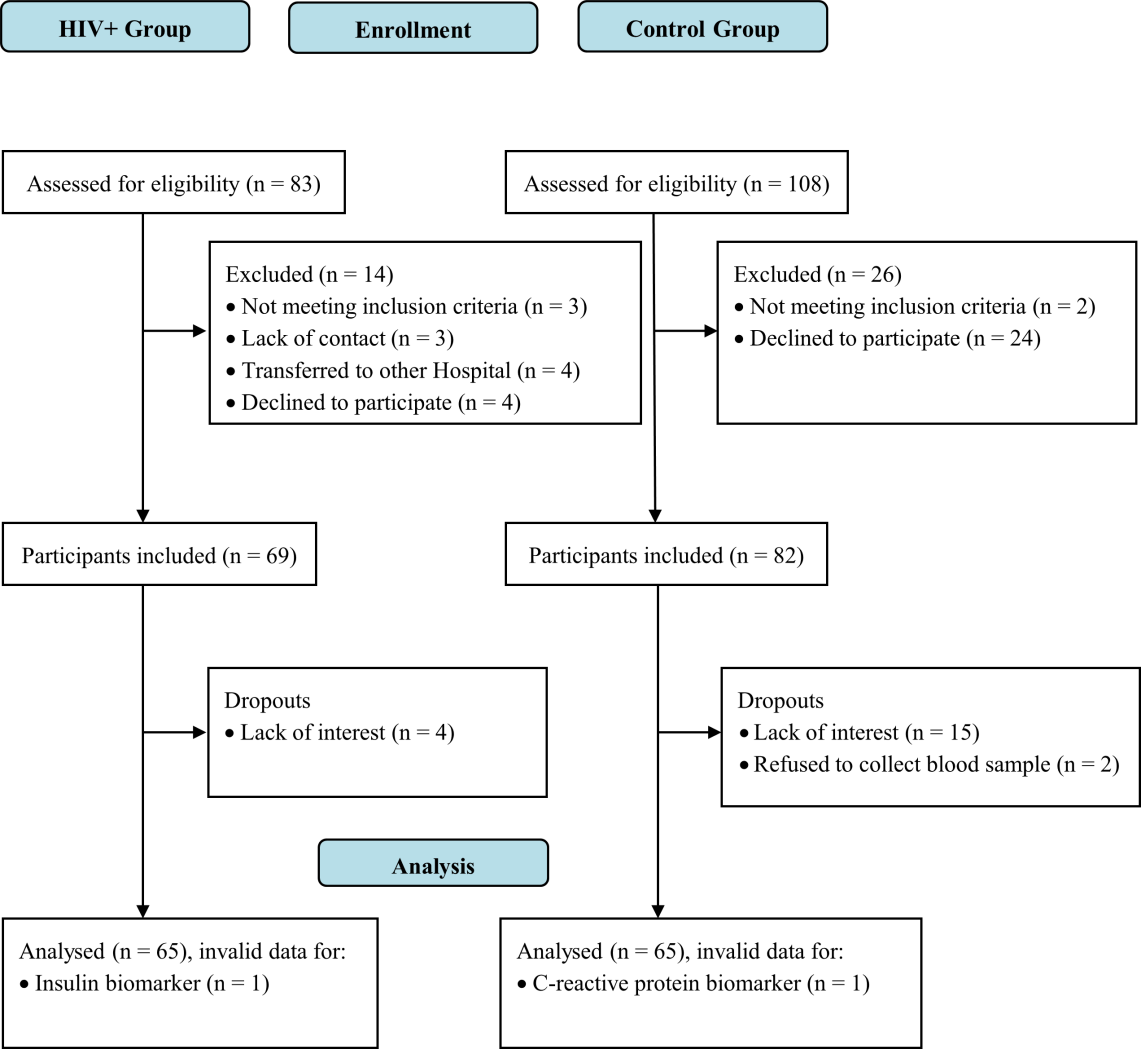
Flowchart of participants. PositHIVe Health Study.

Sixty-five HIV-infected children and adolescents were recruited consecutively during clinical follow-up at the Joana de Gusmão Children's Hospital, a state reference facility in the treatment of HIV infections. Participants met the following inclusion criteria: 1) HIV infection by mother-to-child transmission was verified through medical records; 2) age between 8 and 15 years; 3) the patient was managed by the pediatric infectologist at the hospital regardless of the use of ART; and 4) medical records included clinical and laboratory data. The exclusion criteria were as follows: 1) motor impairment or contraindication to vigorous exercise; 2) impairment of speech, hearing, and/or cognition; 3) diseases that alter body composition, except for HIV infection; and 4) regular use of diuretic medications or immunotherapies. The control group was comprised of a 1:1 ratio and was gender- and age-matched according to the HIV group participants. Recruitment of participants was performed in a non-probabilistic sampling from the health monitoring records of a public school in the same region. The sample size was calculated *a priori* based on type I (α = 0.05) and the power of the study is 80% to identify differences between groups with a large effect size (1.9 and 0.8) on the IMTc [12] and high-density lipoprotein of cholesterol (HDL-c) [15], respectively, and a medium effect size (0.5) on glucose [15]. Details on the eligible, excluded, and lost participants from both groups are in shown in Fig 1.

### Variables

The gender (male vs. female) and skin color (white vs. brown and black) of the participants, income (wages), and education (years of schooling) was recorded. Sexual maturation was self-reported based on the observation of the images of Tanner stages after participants received instructions from a same-gender researcher. For the HIV group, information about ART (untreated vs. non- PI-ART vs. PI-ART), HIV-1 viral load measured by RNA quantification (Abbott RealTime® HIV -1; Abbott, Rungis, France), and CD4 T-cell count measured by flow cytometry (Facscalibur® Multitest; BD Biosciences, San José, CA, USA), were obtained from medical records. The body mass (kg) and height (cm) were measured using a Tanita® electronic scale (BF683W; Arlington Heights, Illinois, USA), and an Alturaexata® stadiometer (Belo Horizonte, Brazil), respectively.

### Cardiovascular risk factors, biomarkers of inflammation, and IMTc

Total and segmented body fat was measured by dual-energy X-ray absorptiometry using a GE® Lunar Prodigy Advance (GE Medical Systems, Madison, WI, USA) with ENCORE software (13.60.033). The equipment was calibrated daily according to the manufacturer, and phantom calibration was performed weekly. A trained researcher performed all tests using standard procedures [19]. The participants wore appropriate clothing without any type of metal and were barefoot. The fat mass index was calculated by the ratio of body fat (kg) to the square of the height (m). The test-retest reproducibility for total body fat was examined in an independent sample that was similar in age and gender (11.6 ± 5.8 and 11.5 ± 5.9 kg; intraclass correlation coefficient = 1.00; 95% confidence interval = 0.99–1.00; n = 10).

Blood samples (15 mL) were collected in the morning after a 10-h fast in a dry tube with gel for the dosages of lipid, glycemic, and inflammatory profile. Samples were centrifuged at 4000 rpm for 10 min to obtain serum. Aliquots were used immediately and other aliquots were stored at −80° C. Total cholesterol and triglycerides were measured using an enzymatic assay. HDL-C was measured by a direct method *in vitro* [20] (Wiener® CB 400i; Rosario, Argentina). Low-density cholesterol (LDL-C) was calculated using the Friedewald equation [21]. Glucose was determined using the oxidase method (Wiener® CB 400i). Insulin was measured by chemiluminescence and the glycated hemoglobin (HbA1c) was determined by liquid chromatography. Homeostatic Model Assessment for Insulin Resistance (HOMA-IR) was calculated [22]. CRP was measured by an immunoturbidimetric assay with latex (Wiener® CB 400i). Quantification of cytokines (IL-6 and TNF-α) was performed using a cytometric bead array human inflammatory cytokine kit (BD® Biosciences, San Jose, CA, USA) for detection of TNF-α and IL-6. The analysis was performed according to the manufacturer's instructions. The concentrations were obtained using the BD FACSVerse® flow cytometer (BD Bioscience®) and analyzed with FCAP Array® software (v3.0).

Systolic (SBP) and diastolic blood pressure (DBP) were measured by an automated oscillometric method using an Omron® device (model HEM 742; Kyoto, Japan) validated for children and adolescents [23]. The procedures for measurement and identification of SBP were performed as recommended by the 4^th^ Task Force Report on High Blood Pressure in Children and Adolescents [24]. Cuffs with appropriate sizes were used. Three repeated measures of SBP and DBP were performed on 2 different days. The mean value was considered for analyses.

IMTc was determined in the right common carotid artery, using as a reference 1 mm of the bulb bifurcation [13] scanned by a Toshiba® ultrasound device (Viamo; Toshiba Medical Systems Corporation, Tokyo, Japan) and a linear transducer with a frequency of 7.5-MHz (model PLT 704ST; Toshiba Medical Systems), with depth of 4 cm and two-dimensional mode. The participants remained in the supine position, with the head at a 45° angle in the opposite direction to the examined side [25]. The three most distinct images of six vascular diastoles were selected. The analysis was performed by an experienced investigator, blind to HIV status, using semi-automated edge detection software (M'Ath® Std; Metris, Argenteuil, France). The mean and maximum values ​​of IMTc were considered for analysis. A calibrated investigator performed the IMTc measurement of the participants and test-retest reproducibility was examined in an independent sample (0.504 ± 0.016 and 0.495 ± 0.014 mm, respectively; intraclass correlation coefficient = 0.94; 95% confidence interval = 0.79–0.98; n = 11).

### Statistical analysis

The mean and standard deviation (SD), median, and interquartile range (IQR) were used to describe the data according to the data distribution. Asymmetry, kurtosis, histograms, and the Kolmogorov–Smirnov test were used to establish normality. Asymmetric data were normalized by a natural logarithm and inverse of the square root. A Student independent *t*-test and the Mann–Whitney U-test were used for comparisons between groups (HIV and control). An analysis of variance (ANOVA) with Bonferroni *post-hoc* test was used to comparison of ART groups. The Kruskal–Wallis test was used for asymmetric variables.

Multiple linear regression analysis was used to test the associations between cardiovascular risk factors (trunk body fat, fat mass index, systolic blood pressure, HOMA-IR, total cholesterol, LDL-c, HDL-c, and triglycerides), inflammation (CRP, IL-6, and TNF-α), and the IMTc with groups (control = 0; HIV untreated = 1; HIV non- PI-ART = 2; HIV PI-ART = 3), adjusted for age, gender, and sexual maturity. Multiple linear regressions were also used to analyze the independent variables associated with the IMTc (outcome) in all children and adolescents infected with HIV. A secondary analysis was tested only in PI-ART patients. Analysis included the following variables: age; sex (vs. female); PI-ART (vs. untreated and non- PI-ART); HIV-1 viral load; CD4 T-cell count; trunk body fat; SBP; glucose; total cholesterol; LDL-C; HDL-C; triglycerides; CRP; IL-6; and TNF-α. All variables were simultaneously inserted into the models and a backward procedure was performed to remove non-significant variables (p > 0.20) until the final model was derived. The post-estimations of goodness-of-fit as multicollinearity and heteroscedasticity of models were performed analyzing, Akaike information criterion, and Bayesian information criterion variance inflation factor, and dispersion of the residuals.

All analyses were conducted on STATA 13.0 (Stata Corporation®, College Station, TX, USA) and GraphPad Prism 5.0 (GraphPad® Software, Inc., San Diego, CA, USA), adopting p < 0.05 for two-tailed tests.

## Results

The study included 130 children and adolescents (HIV group = 65; and control group = 65) between 8.0 and 15.2 years of age. After completion of the investigation steps, one insulin datum and one CRP datum were considered invalid. The socioeconomic and clinical characteristics are summarized in Table 1. The z-score of stature for age was lower in the HIV group and the body mass index for age was lower in the control group. The schooling of the parent / guardian was lower in the HIV group. Moreover, 44 HIV-infected participants (67.7%) had undetectable viral load (<40 copies.mL^−1^). Eleven participants (16.9%) were not on antiretroviral treatment, among these patients 2 abandoned treatment, 1 had a late diagnosis, and 8 had no immunologic and virologic criteria for the introduction of ART according to 2014 Brazilian Ministry of Health Guidelines [26]. Thirty-nine HIV-infected children (60%) are in PI-ART. Most patients in non PI-ART regimen (13 of 15) are exposed to both nucleoside/nucleotide and non-nucleoside reverse transcriptase inhibitors.

**Table 1:** Characteristics of the participants.

	HIV Group (n = 65)	Control Group (n = 65)	t / χ^2^	*P*
Age (years), mean (SD)	12.2 (2.1)	12.1 (1.8)	-0.3071	0.7592 ^a^
Sex, n (%)				
Male	30 (46.2)	30 (46.2)	0.0000	1.0000 ^b^
Female	35 (53.8)	35 (53.8)		
Skin color, n (%)				
White	29 (44.6)	38 (58.5)	2.4947	0.1140 ^b^
Brown / Black	36 (55.4)	27 (41.5)		
Income (wage = $226,12), n (%)				
≤ 2 wage	26 (40.0)	20 (30.8)	1.2112	0.271 ^b^
> 2 wage	39 (60.0)	45 (69.2)		
Education of parent, n (%)				
0–10 years	44 (67.7)	13 (20.0)	30.0240	**<0.0001** ^b^
11–15 years	21 (32.3)	52 (80.0)		
Pubertal Status, n (%)				
Stage I	15 (23.1)	11 (16.9)	5.0510	0.3250 ^b^
Stage II	20 (30.7)	19 (29.2)		
Stage III	19 (29.3)	22 (33.9)		
Stage IV	8 (12.3)	13 (20.0)		
Stage V	3 (4.6)	0 (0.0)		
z-score Height/Age (SD), mean (SD)	-0.53 (1.12)	0.24 (0.91)	4.3806	**<0.0001** ^a^
z-score BMI/Age (SD), mean (SD)	-0.20 (1.01)	-0.66 (0.85)	-2.8414	**0.0052** ^a^
CD4^+^ (cells.mm^-3^), median (IQR)	819.0 (575.0; 1091.0)	-	-	-
CD4+ (%), median (IQR)	31.2 (24.9; 37.6)	-	-	-
Viral Load (log), median (IQR)	1.60 (1.60; 2.57)	-	-	-

SD: standard deviation; BMI: body mass index; IQR: interquartile range. Both groups were paired by age and sex. Data in bold indicate significance.

^a^ Unpaired Student t-test.

^b^ Chi-square test.

HIV-infected children and adolescents were similar to controls with respect to total body fat, trunk body fat, fat mass index, SBP, DBP, insulin, HOMA-IR, and total cholesterol; however, the HIV group had higher values of glucose, triglycerides, LDL-C, total cholesterol (TC) / HDL-C, LDL-C / HDL-C, CRP, IL-6, and TNF-α when compared to the control group. HDL-C and HbA1c were lower in the HIV group. The mean and maximum IMTc were significantly increased in the HIV group compared to the control group (Table 2). No participants used hypoglycemic agents, anti-hypertensives, statins, or fibrates. Two participants in the control group used vitamins (C and D) and one in the HIV group made use of the B complex. Nobody admitted to alcohol or tobacco use in the last year.

**Table 2:** Cardiovascular risk factors, inflammatory markers and IMTc.

	HIV Group (n = 65)	Control Group (n = 65)	t / z	*P*
**Morphologic and Metabolic**	**Mean (SD) / Median (IQR)**			
Body Fat (%)	21.5 (8.9) ^a^	21.4 (7.9)	-0.0751	0.940
Trunk Body Fat (%)	20.3 (9.2) ^a^	20.7 (8.1)	0.3009	0.764
Fat Mass Index (kg.cm^-2^) †	3.8 (2.0) ^a^	3.47 (1.4)	-0.817	0.401
Glucose (mg.dL^-1^)	87.9 (10.8) ^a^	75.9 (6.3)	-7.7089	**<0.001**
Insulin (μUI.mL^-1^) *	6.4 (3.8) ^a^	5.9 (3.2)	-0.5572	0.578
HOMA-IR*	1.43 (0.98) ^a^	1.11 (0.68)	-1.6968	0.092
HbA1c (%)	4.9 (0.4) ^a^	5.1 (0.3)	3.0736	**0.003**
Total cholesterol (mg.dL^-1^)	168.6 (43.6) ^a^	161.3 (28.0)	-1.1438	0.255
LDL-c (mg.dL^-1^)	94.7 (35.8) ^a^	79.5 (30.1)	-2.6234	**0.010**
HDL-c (mg.dL^-1^)	53.7 (14.2) ^a^	69.4 (12.5)	6.7205	**<0.001**
Total cholesterol/HDL-c *	3.3 (0.9) ^a^	2.4 (0.7)	-6.7986	**<0.00**
LDL-c/HDL-c †	1.8 (0.7) ^a^	1.2 (0.6)	-5.5296	**<0.001**
Triglycerides (mg.dL^-1^) *	101.2 (51.6) ^a^	61.6 (32.9)	-5.6487	**<0.001**
**Inflammatory Markers**				
CRP (mg.L^-1^) *	1.6 (1.2; 2.7) ^b^	1.1 (0.3; 2.5)	-2.713	**0.007**
IL-6 (pg.mL^-1^) †	1.42 (0.97; 2.30) ^b^	0.01 (0.01; 1.04)	-5.300	**<0.001**
TNF-α (pg.mL^-1^) †	0.49 (0.07; 1.00) ^b^	0.01 (0.01; 0.01)	-6.631	**<0.001**
**Vascular Measurements**				
Systolic Blood Pressure (mmHg)	103.2 (8.3) ^a^	105.5 (10.0)	1.2922	0.199
Diastolic Blood Pressure (mmHg)	61.1 (8.3) ^a^	63.5 (10.1)	1.5003	0.136
IMTc mean (mm)	0.526 (0.100) ^a^	0.490 (0.044)	-2.6489	**0.009**
IMTc max (mm)	0.615 (0.112) ^a^	0.581 (0.057)	-2.1914	**0.030**

SD: standard deviation; IQR: interquartile range; BF: body fat; HOMA-IR: Homeostatic Model Assessment for Insulin Resistance; HbA1c: glycated hemoglobin; LDL-c: low density lipoprotein; HDL-c: high density lipoprotein; CRP: C-reactive protein; IL-6: interleukin-6; TNF-α: tumor necrosis factor alpha. IMTc: carotid artery intima-media thickness. Data in bold indicate significance.

^a^ Mean and standard deviation (Unpaired Student’s t-test)

^b^ Median and interquartile range (Mann-Whitney U test).

* ln transformation

† square root transformation.

HIV-infected children and adolescents were compared in the ART groups (Table 3) and were similar with respect to age, gender, and sexual maturation. PI-ART patients presented high values of TC / HDL-C, total cholesterol, and triglycerides. The HIV-1 viral load and TNF-α were higher and the CD4 T-cell count was lower than untreated patients compared to both ART regimens.

**Table 3:** Comparison of HIV-infected children and adolescents in different ART regimens.

	Not Treated (n = 11)	Non PI-ART (n = 15)	PI-ART (n = 39)	F / χ^2^	*p*
	Mean (SD) ^a^ / Median (IQR) ^b^ / Absolute frequency ^c^				
Age (years)	12.5 (1.9) ^a^	12.7 (1.7)	12.0 (2.3)	1.629	0.4428
Sex (male/ female)	5/6 ^c^	6/9	19/20	0.3339	0.846
Pubertal Status (I/ II/ III/ IV/ V)	4/3/2/1/1 ^c^	2/3/6/3/1	9/14/11/4/1	5.3863	0.716
**Morphologic and Metabolic**					
Body Fat (%)	25.8 (6.5) ^a^	22.9 (10.4)	19.8 (8.6)	2.28	0.1104
Trunk Body Fat (%)	24.3 (7.0) ^a^	22.3 (11.0)	18.3 (8.7)	2.40	0.0995
Fat Mass Index (kg.cm^-2^) †	4.53 (1.77) ^a^	4.21 (2.51)	3.45 (1.82)	1.86	0.1643
Glucose (mg.dL^-1^)	84.7 (8.0) ^a^	89.7 (9.9)	88.1 (11.8)	0.69	0.5043
Insulin (μUI.mL^-1^) *	6.0 (3.7) ^a^	6.8 (3.5)	6.3 (4.1)	0.98	0.3823
HOMA-IR*	1.29 (0.89) ^a^	1.56 (0.82)	1.42 (1.06)	0.94	0.3965
HbA1c (%)	5.1 (0.4) ^a^	4.9 (0.4)	5.1 (1.3)	0.93	0.4000
Total Cholesterol (mg.dL^-1^)*	143.3 (38.6) ^a^	155.1 (40.8)	180.9 (42.3) §	5.59	**0.0059**
LDL-c (mg.dL^-1^) †	83.9 (32.4) ^a^	82.3 (36.4)	102.5 (35.1)	2.99	0.0575
HDL-c (mg.dL^-1^)	44.8 (5.2) ^a^	57.5 (12.8)	54.7 (15.6)	2.95	0.0596
Total cholesterol/HDL-c *	3.2 (0.8) ^a^	2.8 (0.8)	3.5 (0.9) **	4.41	**0.0162**
LDL-c/HDL-c †	1.9 (0.7) ^a^	1.5 (0.8)	1.9 (0.7)	2.93	0.0607
Triglycerides (mg.dL^-1^)*	72.7 (33.7) ^a^	76.7 (30.8)	118.7 (55.2) §	6.65	**0.0024**
**Inflammatory markers**					
CRP (mg.L^-1^)	1.2 (0.4; 4.9)^b^	1.9 (1.0; 6.4)	1.8 (1.2; 2.9)	4.256	0.1191
IL-6 (pg.mL^-1^)	1.45 (1.08; 2.30)^b^	1.77 (0.90; 2.85)	1.42 (0.66; 2.19)	0.857	0.6513
TNF-α (pg.mL^-1^)	0.95 (0.64; 1.44)^b^	0.39 (0.20; 1.66)	0.34 (0.01; 0.69) ††	7.030	**0.0297**
**Vascular Measurements**					
Systolic Blood Pressure (mmHg)	97.5 (10.6) ^a^	102.4 (9.0)	105.2 (9.1)	3.02	0.0562
Diastolic Blood Pressure (mmHg)	61.5 (8.7) ^a^	58.2 (6.5)	62.1 (8.7)	1.22	0.3031
IMTc mean (mm)	0.500 (0.041) ^a^	0.547 (0.127)	0.525 (0.101)	0.66	0.5190
IMTc max (mm)	0.579 (0.045) ^a^	0.639 (0.133)	0.616 (0.116)	0.89	0.4158
**Clinical and Laboratorial Data**					
Viral load (log)	3.46 (2.70; 3.94) ^b^	1.60 (1.60; 1.60) ††	1.60 (1.60; 1.60) ††	12.137	**0.0023**
Undetectable viral load (frequency)	2/11 ^c^	12/15	30/39	14.887	**0.0010**
Lymphocytes T CD4^+^ (cells.mm^-3^)	534.4 (223.3) ^a^	790.1 (208.2)	974.7 (390.5) ††	7.88	**0.0009**
Lymphocytes T CD4+ (%)	20.2 (9.8) ^a^	31.1 (6.8) ††	32.6 (7.6) ††	11.00	**0.0001**

SD: standard deviation; IQR: interquartile range; BF: body fat; HOMA-IR: Homeostatic Model Assessment for Insulin Resistance; HbA1c: glycated hemoglobin; LDL-c: low density lipoprotein; HDL-c: high density lipoprotein; CRP: C-reactive protein; IL-6: interleukin-6; TNF-α: tumor necrosis factor alpha; IMTc: carotid artery intima-media thickness. Significant differences are in bold.

^a^ Mean and standard deviation (ANOVA with Bonferroni Post-hoc).

^b^ Median and interquartile range (Kruskal-Wallis test).

^c^ Absolute frequency (Chi-square test).

* ln transformation

†square root transformation.

§ difference from not treated and PI-ART

** difference from non PI-based ART

†† different from not treated.

**Fig 2:**
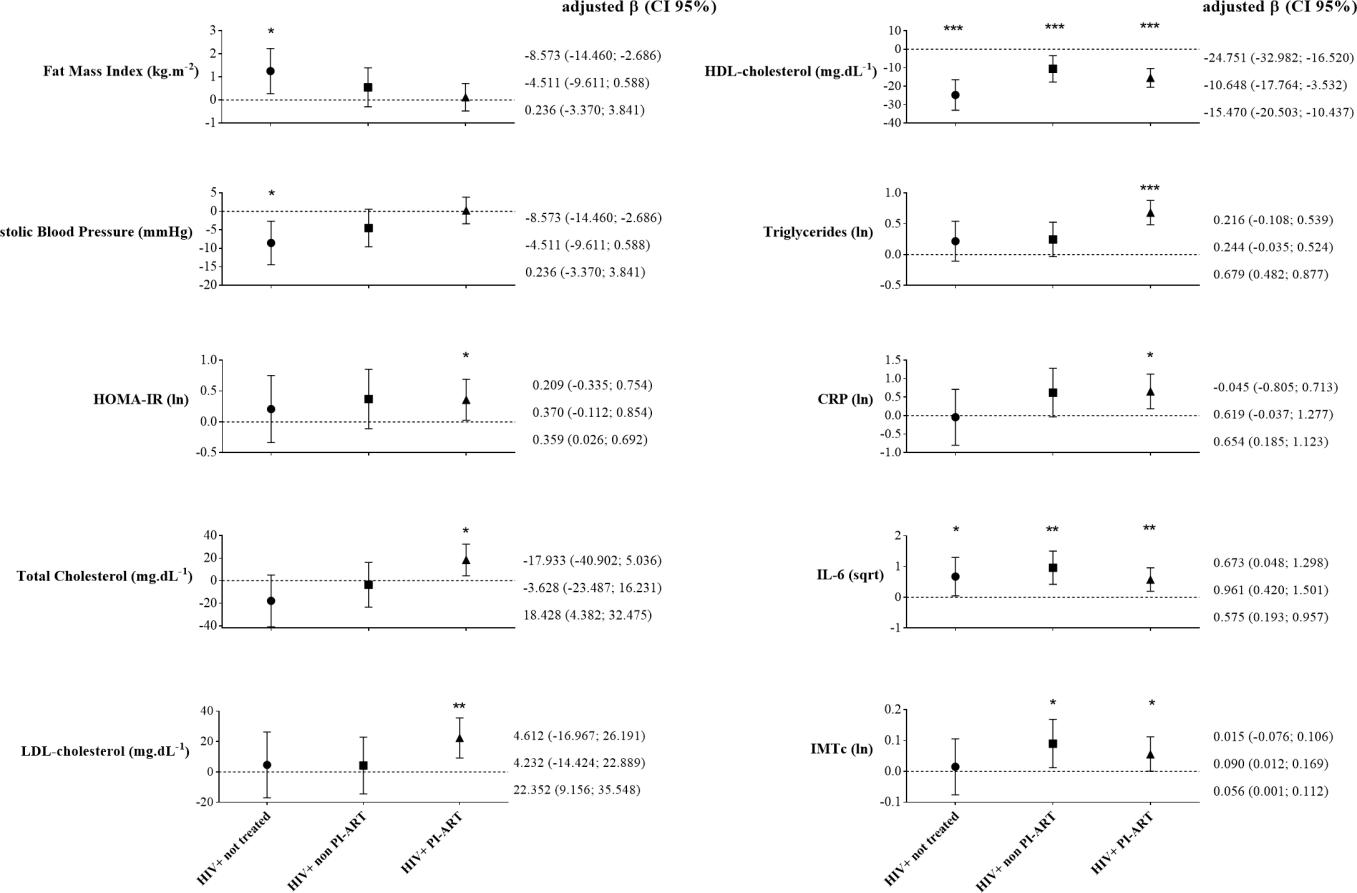
Beta regression coefficients of group associations with cardiovascular risk factors, inflammation and intimal-media thickness. Independent variable: Control = 0 (reference); HIV-infected not treated = 1; HIV-infected non PI-ART = 2; HIV-infected PI-ART = 3. * p-value < 0.05; ** < 0.01; *** <0.001. All models were adjusted by age, sex and pubertal status.

Fig 2 and S1 Table present the distribution of cardiovascular risk factors, biomarkers of inflammation, and the IMTc according to the ART regimen. When compared to the control group, HIV infection in PI-ART patients was associated with an increase in total cholesterol (18.4 mg.dL^−1^), LDL-C (22.6 mg.dL^−1^), triglycerides (0.67 natural log), HOMA-IR (0.35), IL-6 (0.57 square root), CRP (0.65 natural log), and IMTc (0.05 natural log). Likewise, HIV infection in non-PI-ART patients was associated with a lower HDL-C (−10.6 mg.dL^−1^) and a higher IL-6 (0.96 of the square root) and IMTc (0.09 of the natural logarithm) when compared to control group. HIV-infected untreated patients was associated with an increase in fat mass index (1.25 kg.m^−2^), IL-6 (0.67 square root), a reduction in SBP (8.6 mmHg) and HDL-C (24.7 mg.dL^−1^) compared to control group. Since, viral load could influence inflammatory mediators, we compared CRP, IL-6 and TNF-α levels between patients who achieved undetectable viral and patients with > 40 copies.ml^-1^. No statistical differences were found in inflammatory mediators analyzed (S2 Table).

The increased SBP and decreased TNF-α were associated with increased IMTc, independent of IL-6, glucose, and the PI-ART regimen in HIV-infected children and adolescents (Table 4). The final model explained 16% of the variability of the IMTc (p = 0.0080; F = 3.48). A complementary analysis adjusting for BMI, ethnicity, and income did not change results. Among the HIV-infected children and adolescents on PI-ART, male gender, lower of trunk body fat, increased glucose, and IL-6 were associated with increased IMTc independent of age and SBP (Table 4). The final model explained 28% of the IMTc variability (p = 0.0100; F = 3.42).

**Table 4:** Multiple linear regression analysis of the independent predictors of IMTc (ln).

	HIV-infected participants (N = 65)			HIV-infected participants in PI-ART (N = 39)		
	Model 1* (R^2^ = 0.16)			Model 2** (R^2^ = 0.28)		
	β (CI 95%)	β _standardized_	p-value	β (CI 95%)	β _standardized_	p-value
Age (years)	-	-	NS	0.023 (-0.002; 0.047)	0.293	0.066
Male (vs. female)	-	-	NS	**-0.186 (-0.319; -0.053)**	**-0.527**	**0.008**
PI-ART (vs. not treated/ non- PI-ART)	-0.056 (-0.141; 0.028)	-0.163	0.187	NA	NA	NA
Trunk Body Fat (%)	-	-	NS	**-0.011 (-0.019; -0.003)**	**-0.559**	**0.006**
Systolic Blood Pressure (mmHg)	**0.006 (0.002; 0.011)**	**0.360**	**0.004**	0.004 (-0.002; 0.010)	0.209	0.173
Glucose (mg.dL^-1^)	0.003 (-0.001; 0.007)	0.228	0.054	**0.005 (0.001; 0.009)**	**0.302**	**0.046**
IL-6 (pg.mL^-1^)	0.011 (-0.003; 0.025)	0.293	0.115	**0.017 (0.001; 0.032)**	**0.308**	**0.039**
TNF-a (g.mL^-1^)	**-0.033 (-0.062; -0.003)**	**-0.420**	**0.029**	-	**-**	NS

* p = 0.0080; F = 3.48; root mean square error = 0.156; mean VIF = 1.71; BIC = -305.070; AIC*n = -0.753.

** p = 0.0100; F = 3.42; root mean square error = 0.152; mean VIF = 1.23; BIC = -157.709; AIC*n = -0.721.

R^2^: coefficient of determination. β: regression coefficient (unstandardized). NS: no statistical significance at p < 0.20. NA: not applicable. PI-ART: antiretroviral therapy with protease inhibitors; IL-6: interleukin-6; TNF-α: tumor necrosis factor alpha. IMTc: carotid artery intima-media thickness. All variables were tested simultaneously and variables were removed in backward procedure starting with the higher p value until p < 0.20 and significant model p < 0.05. Significant predictors (p < 0.05) are in bold.

## Discussion

The main finding of the present study was HIV-infected children and adolescents had profiles potentially associated with premature atherosclerosis due to inflammation, elevated IMTc, higher atherogenic lipid levels, and higher blood glucose levels. HIV infection was associated with low HDL-C and high IL-6 levels regardless of the use of ART. PI-ART was associated with dyslipidemia, insulin resistance, high CRP, and increased IMTc. In this study, SBP and TNF-α were associated with elevated IMTc in HIV-infected children and adolescents. In patients using PI-ART, male gender, trunk body fat, glucose, and IL-6 were predictors of increased IMTc.

The difference found in IMTc among HIV-infected children and adolescents compared to controls (36 μm) corroborates previous evidence to a variable degree (10–130 μm) [12–15]. According to the data of Weberruß et al. [27], boys 8 years of age had an 18 μm difference in the IMTc median compared to boys 18 years of age. Therefore, the difference between groups observed in our study represents twice the corresponding 10 years of thickening in healthy children and adolescents [27]. Our study was similar to the previous in variability of ART schemes [13, 14], in the majority (76.4%–84%) with undetectable viral loads [14, 15], and in the presence of cardiovascular risk factors [12–15]. Studies that did not find significant differences in the IMTc [11, 28] may have diverged due to variability of the clinical condition of patients, type of ART instituted at the stage of studies, magnitude of cardiovascular risk factors, sample size, and heterogeneity of the technique to measure the IMTc. The increase in the IMTc represents a cumulative damage in the endothelium structure, classically demonstrated by the presence and severity of cardiovascular risk factors [29]. However, in HIV-infected children and adolescents the increased risk of atherosclerotic disease occurs due to exposure to chronic inflammation from the earliest stages of the disease due to mother-to-child transmission, which corroborates the finding of significant IMTc among other cardiovascular manifestations [30]. The clinical implication of the observed IMTc is uncertain. However, the IMTc measurement is a noninvasive, sensitive, and reproducible technique [25], also, a recognized surrogate marker of atherosclerosis and nonatherosclerotic compensatory remodeling with largely medial hypertrophy [31]. Thus, the detectable structural changes in the intimal and medial layers at the second decade of life, when atherosclerotic plaque isn’t evident, could preclude early development of morbidity and mortality due to cardiovascular diseases, especially in young people with severe abnormalities of individual risk factors [32].

SBP and TNF-α were independently associated with increased IMTc, accounting for 16% of the variability in this study. High blood pressure is a classical cardiovascular risk factor that contributes to the thickening by the remodeling of the endothelium walls due to the shear forces that affect the vascular tree. SBP was previously shown to be a predictor of IMTc [27] and associated with fatty streaks and fibrous plaques in the coronary arteries and aorta [9] in children and adolescents of the general population and in children with primary hypertension [33]. The prediction of IMTc by the decrease in TNF-α seems controversial and may be attributed to participants with more advanced disease and higher IMTc at the moment also had lower inflammation due to the introduction PI-ART as a second-line treatment. However, it is difficult to explain the unexpected association due to circumstances of the cross-sectional design, which includes inability to infer causality and does not take in account patient history (e.g. infection-related hospitalization) that can predict atherosclerosis [34]. In the exclusive analysis of HIV-infected children and adolescents using PI-ART, male gender, lower trunk body fat, higher glucose, and IL-6 were independent predictors of IMTc, accounting for 28% of the variability. Blood glucose has been recognized as a risk factor for atherosclerotic disease in the general population due to the glycosylation process of LDL-C [35]. Indeed, in the subclinical context, an increase in the IMTc was observed in adolescents with diabetes mellitus type I and II compared to controls [36]. The inverse association of trunk body fat with IMTc in patients using PI-ART could be an explained, in part, by body fat redistribution as result of complex interactions of symptomatic HIV infection, host characteristics, genetic and lifestyle factors. Gender, as a predictor of IMTc, corroborates an earlier investigation [37] and can be explained by the fact that males show a trend towards higher IMTc compared to females [27]. Increased IL-6, which predicts the increase in IMTc, may be a consequence of lymphocyte and dendritic cell activation, mucosal damage, endothelial damage, metabolic changes, and / or factors related to HIV replication [38], even in HIV-infected people that achieve an undetectable viral load [39].

Results of our study indicated HIV infection was associated with elevated levels of IL-6 and reduced HDL-C regardless of antiretroviral use (treated or untreated) or type (Non PI-ART or PI-ART), Both parameters may contribute to premature atherosclerosis and mortality through chronic inflammation, thrombotic activity, and damage to the endothelial structure [10, 38, 40]. Low levels of HDL-C (−24.7 mg.dL^−1^) associated with HIV infection in untreated children and adolescents suggests HIV *per se* as a risk factor for dyslipidemia, which corroborates previous studies with therapy-naive HIV-infected children and adolescents [41, 42]. Furthermore, HDL-C can be considered a negative acute phase protein that is reduced in chronic inflammation. In a representative sample from the same city of the present study, Giuliano et al. [43] demonstrated an inverse association with CRP, possibly influenced by the shift of metabolic pathways to the synthesis of acute phase proteins over lipoprotein production.

We found increased IMTc were associated with HIV infection in PI-ART and non- PI-ART regimens. These findings corroborate previous studies in HIV-infected children and adolescents [12–15], mainly of the patients in using PI's [12, 15]. Furthermore, a meta-analysis showed an increased IMTc in 63 μm among ART comparison (HIV patients in ART versus HIV patients naïve to ART) and 33 μm among PI-ART use comparison (HIV in PI-ART versus HIV in non- PI-ART regime) [44]. The evidence supports a deleterious effect of HIV *per se* [15, 30], and this is true in both ART groups because are composed by symptomatic HIV-infected patients who met the virologic and immunologic criteria for introduction of ART, according to the Brazilian Ministry of Health [26]. In this group, a NRTI was administered as first-line treatment and the PIs were secondarily introduced to patients who were severely symptomatic or had a very high viral load. Based on the guidelines, asymptomatic pediatric patients remain without antiretroviral medication [26]. This scenario may justify an association between ART and IMTc, but conceals a composition of the groups that was naturally defined by evolution and severity of the disease. Thus, it is necessary to keep the focus that chronic inflammation imposed by HIV is the central mechanism that may cause an increase the risk of cardiac disease [45].

In addition, HIV-infection in PI-ART was associated with elevated total cholesterol, LDL-C, triglycerides, HOMA-IR, and CRP levels. Although we have found these results with small samples size in HIV-infected ART groups, previous studies with large samples have already evidenced these associations [2, 4, 46]. Dyslipidemia induced by PI-ART is associated with inhibition of the expression of LDL-C related receptors, which results in elevated LDL-C in the circulation [47]. Moreover, binding with LDL-C related receptors interferes in the formation of the lipase complex and reduces storage of triglycerides, with a subsequent increase in the circulation [47], the mechanism of which may be increased synthesis of triglycerides by hepatocytes and lipoatrophy [47]. High total cholesterol, CT / HDL-C, and triglycerides in HIV-infected children and adolescents in PI-ART have been previously observed [4, 5, 13]. Dyslipidemia appears to be even more manifest in the use of Ritonavir [2, 3], which was used (lopinavir boosted with ritonavir) in the PI-ART of most children and adolescents in our study. Insulin resistance has been explained by inhibition of GLUT-4 [48], although changes in carbohydrate metabolism appear to be mediated by lipohypertrophy [49], lipoatrophy, and dyslipidemias due to mitochondrial toxicity in the liver and muscles [47]. Elevations in HOMA-IR [2, 49] and insulinemia [2] are more often associated with PI-ART, although other studies have not reported this association [4, 13, 41]. The higher levels of CRP in HIV-infected patients in PI-ART (Fig 2) are consistent with the metabolic and cardiovascular deviations, as well as the severity of HIV infection and the IMTc. The SMART study demonstrated that a high value of CRP predicts all-cause mortality in HIV-infected adults, even when viral suppression was achieved [10].

The comparison between the HIV group and controls revealed that abnormal values of LDL-C, TC / HDL-C, LDL-C / HDL-C, triglycerides, and HDL-C were found, although the differences may have no clinical significance given the magnitude and variation of the means. Conversely, inflammatory markers (i.e., CRP, IL-6, and TNF-α) were highlighted and elevated in the HIV group compared to the controls, which characterizes the state of chronic inflammation. Although there is a lack of consensus in the literature, as some studies have reported elevations in CRP [11–13], IL-6 [16, 50], and TNF-α [50], while others have not [2, 11, 15, 18], the chronic inflammation was consistent with increased IMTc in the HIV group compared to controls. The concern with HIV-infected children and adolescents with long-term exposure to ART lies in the substantial increase in the risk of developing atherosclerotic disease prematurely.

The limitations of this study include cross-sectional design that does not allow inferring the causality of the effect of HIV infection and ART regimens in the outcome; as the inability to evaluate change over time on the variables. The sample size was calculated to find differences in outcomes between HIV and control group; thus, other analysis may are underpowered and the results have limited generalization. The absence of additional markers of atherosclerosis (i.e., LDL-C and HDL-C particles, markers of coagulation, and activation and adhesion to the endothelium) was another limitation. The strength of the study includes a representative sample of HIV-infected patients (78% of the patients served in a pediatric referral center) with an age range that included puberty with the presence of a sex- and age-matched control group and the blinded analysis for of HIV infection and the ART regimen of IMTc by an independent observer.

We conclude that HIV-infected children and adolescents may be at risk for premature atherosclerosis due to inflammation, elevated IMTc, higher atherogenic lipid levels, and higher blood glucose levels. Patients using PI-ART were associated with dyslipidemia, insulin resistance, chronic inflammation, and increased IMTc. SBP and TNF-α were independent predictors of IMTc in the model that comprised all HIV-infected patients. There were independent predictors of the IMTc in patients in PI-ART, such as male gender, reduction of trunk body fat, and increases in glucose and IL-6. Future studies with larger sample size need to evaluate the prospective associations between cardiovascular risk factors, inflammatory mediators and IMTc. The monitoring of these health markers is important in pediatric patient care, in the mid- and long-term, on the perspective of prevention of atherosclerotic disease, even more at present due to the increase in the survival of the HIV-infected population. Furthermore, there is a need to formulate interventions, with lifestyle changes and drug therapies, with the potential to improve lipid profile, mitigate inflammation, and reduce cardiovascular risk in HIV-infected children and adolescents.

## Supporting information

S1 Table Multiple linear regression coefficients for cardiovascular risk factors, inflammation and intimal-media thickness associated with HIV-infection. Legend: HOMA-IR: Homeostatic Model Assessment for Insulin Resistance; LDL: low density lipoprotein; HDL: high density lipoprotein; CRP: high-sensitive C-reactive protein; IL-6: interleukin-6; TNF-α: tumor necrosis factor alpha; IMTc: carotid artery intima-media thickness.
Independent variable: Control = 0 (reference); HIV-infected not treated = 1; HIV-infected non- PI-ART = 2; HIV-infected PI-ART = 3.
† ln and †† square root transformation. * Significant at least <0.05; ** p-value of HIV-infection. All models were adjusted by age, sex and pubertal status.
(DOCX)

S2 Table Crude and adjusted comparison of inflammatory markers between patients had achieved undetectable viral load (HIV RNA ≤ 40 copies.ml) and patients with > 40 copies/ml. Legend: * ln transformation; † square root transformation. ^a^ Adjusted for age, sex, maturity, body max index and use of protease inhibitors in antiretroviral treatment.
CRP: C-reactive protein; IL-6: interleukin-6; TNF-α: tumor necrosis factor alpha.
(DOCX)

S1 Dataset PositHIVe Health Study data. These are raw data and transformed variables from HIV-infected patients and healthy controls used for all analyses. (DTA).
(RAR)

## Abbreviations

ART: antiretroviral therapy
CRP: C-reactive protein
DBP: diastolic blood pressure
HbA1c: glycated hemoglobin
HDL-C: high-density lipoprotein of cholesterol
HIV: human immunodeficiency virus
HOMA-IR: Homeostatic Model Assessment for Insulin Resistance IL-6, interleukin-6
IMTc: carotid intima-media thickness
LDL-C: low-density lipoprotein of cholesterol
NRTI: nucleoside/nucleotide reverse transcriptase inhibitor
PI: protease inhibitor SBP, systolic blood pressure
TC: total cholesterol
TNF-α: tumor necrosis factor-alpha
