# Supplementary material for: Dyslipidemia, chronic inflammation, and subclinical atherosclerosis in children and adolescents infected with HIV: The PositHIVe Health Study
Source: PLoS One. 2018 Jan 10;13(1):e0190785. doi: 10.1371/journal.pone.0190785 (PMC5761890; doi:10.1371/journal.pone.0190785)
Supplement: S1 Table — Legend: HOMA-IR: Homeostatic Model Assessment for Insulin Resistance; LDL: low density lipoprotein; HDL: high density lipoprotein; CRP: high-sensitive C-reactive protein; IL-6: interleukin-6; TNF-α: tumor necrosis factor alpha; IMTc: carotid artery intima-media thickness. Independent variable: Control = 0 (reference); HIV-infected not treated = 1; HIV-infected non- PI-ART = 2; HIV-infected PI-ART = 3. † ln and †† square root transformation. * Significant at least <0.05; ** p-value of HIV-infection. All models were adjusted by age, sex and pubertal status. (DOCX) [file pone.0190785.s001.docx]

**S1 Table. Multiple linear regression coefficients for cardiovascular risk factors, inflammation and intimal-media thickness associated with HIV-infection.**

| **Outcomes** |  |  |  |  |
| --- | --- | --- | --- | --- |
|  | **Crude β (CI 95%)** | **Adjusted β (CI 95%)** | **β_standardized_** | **p-value**** |
| Trunk Body Fat (%) |  |  |  |  |
| HIV+ not treated | 3.611 (-1.904; 9.126) | 4.165 (-0.505; 8.836) | 0.134 | 0.080 |
| HIV+ non PI-ART | 1.570 (-3.275; 6.416) | 0.824 (-3.214; 4.863) | 0.030 | 0.687 |
| HIV+ PI-ART | -2.384 (-5.810; 1.042) | -1.883 (-4.740; 0.973) | -0.100 | 0.194 |
| Fat Mass Index (kg.m^-2^) |  |  |  |  |
| HIV+ not treated | 1.054 (-0.066; 2.176) | 1.250 (0.272; 2.227) | 0.199 | **0.013** |
| HIV+ non PI-ART | 0.736 (-0.249; 1.721) | 0.550 (-0.295; 1.395) | 0.100 | 0.200 |
| HIV+ PI-ART | -0.023 (-0.719; 0.674) | 0.121 (-0.476; 0.719) | 0.032 | 0.689 |
| Systolic Blood Pressure (mmHg) |  |  |  |  |
| HIV+ not treated | -8.013 (-14.265; -1.761) * | -8.573 (-14.460; -2.686) | -0.244 | **0.005** |
| HIV+ non PI-ART | -3.113 (-8.609; 2.380) | -4.511 (-9.611; 0.588) | -0.147 | 0.082 |
| HIV+ PI-ART | -0.265 (-4.156; 3.626) | 0.236 (-3.370; 3.841) | 0.011 | 0.897 |
| HOMA-IR (ln) |  |  |  |  |
| HIV+ not treated | 0.179 (-0.370; 0.729) * | 0.209 (-0.335; 0.754) | 0.068 | 0.448 |
| HIV+ non PI-ART | 0.442 (-0.054; 0.938) | 0.370 (-0.112; 0.854) | 0.134 | 0.131 |
| HIV+ PI-ART | 0.308 (-0.033; 0.650) | 0.359 (0.026; 0.692) | 0.193 | **0.035** |
| Total Cholesterol (mg. dL^-1^) |  |  |  |  |
| HIV+ not treated | -17.989 (-40.714; 4.736) | -17.933 (-40.902; 5.036) | -0.136 | 0.125 |
| HIV+ non PI-ART | -6.128 (-26.094; 13.838) | -3.628 (-23.487; 16.231) | -0.032 | 0.718 |
| HIV+ PI-ART | 19.690 (5.571; 33808) * | 18.428 (4.382; 32.475) | 0.231 | **0.011** |
| LDL-cholesterol (mg. dL^-1^) |  |  |  |  |
| HIV+ not treated | 4.395 (-16.616; 25.406) | 4.612 (-16.967; 26.191) | 0.038 | 0.673 |
| HIV+ non PI-ART | 2.752 (-15.707; 21.213) | 4.232 (-14.424; 22.889) | 0.040 | 0.654 |
| HIV+ PI-ART | 23.032 (9.979; 36.086) * | 22.352 (9.156; 35.548) | 0.304 | **0.001** |
| HDL-cholesterol (mg. dL^-1^) |  |  |  |  |
| HIV+ not treated | -24.627 (-33.119; -16.136) * | -24.751 (-32.982; -16.520) | -0.446 | **<0.0001** |
| HIV+ non PI-ART | -11.912 (-19.373; -4.452) * | -10.648 (-17.764; -3.532) | -0.220 | **0.0004** |
| HIV+ PI-ART | -14.779 (-20.054; -9.504) * | -15.470 (-20.503; -10.437) | -0.459 | **<0.0001** |
| Triglycerides (ln) † |  |  |  |  |
| HIV+ not treated | 0.225 (-0.090; 0.540) | 0.216 (-0.108; 0.539) | 0.106 | 0.189 |
| HIV+ non PI-ART | 0.264 (-0.013; 0.541) | 0.244 (-0.035; 0.524) | 0.139 | 0.086 |
| HIV+ PI-ART | 0.672 (0.476; 0.868) * | 0.679 (0.482; 0.877) | 0.553 | **<0.0001** |
| CRP (ln) † |  |  |  |  |
| HIV+ not treated | -0.035 (-0.768; 0.699) | -0.045 (-0.805; 0.713) | -0.011 | 0.906 |
| HIV+ non PI-ART | 0.612 (-0.034; 1.257) | 0.619 (-0.037; 1.277) | 0.173 | 0.064 |
| HIV+ PI-ART | 0.662 (0.203; 1.122) * | 0.654 (0.185; 1.123) | 0.261 | **0.007** |
| IL-6 (sqrt) †† |  |  |  |  |
| HIV+ not treated | 0.748 (0.134; 1.363) * | 0.673 (0.048; 1.298) | 0.186 | **0.035** |
| HIV+ non PI-ART | 0.932 (0.393; 1.472) * | 0.961 (0.420; 1.501) | 0.305 | **0.001** |
| HIV+ PI-ART | 0.610 (0.228; 0.991) * | 0.575 (0.193; 0.957) | 0.262 | **0.004** |
| TNF-α (sqrt) †† |  |  |  |  |
| HIV+ not treated | 0.592 (-0.081; 1.266) | 0.562 (-0.131; 1.256) | 0.149 | 0.111 |
| HIV+ non PI-ART | 0.606 (0.014; 1.199) * | 0.571 (-0.029; 1.170) | 0.173 | 0.062 |
| HIV+ PI-ART | 0.218 (-0.201; 0.636) | 0.227 (-0.197; 0.651) | 0.099 | 0.291 |
| IMTc mean (ln) † |  |  |  |  |
| HIV+ not treated | 0.022 (-0.066; 0.111) | 0.015 (-0.076; 0.106) | 0.030 | 0.742 |
| HIV+ non PI-ART | 0.092 (0.015; 0.170) * | 0.090 (0.012; 0.169) | 0.208 | **0.024** |
| HIV+ PI-ART | 0.057 (0.002; 0.112) * | 0.056 (0.001; 0.112) | 0.186 | **0.047** |

HOMA-IR: Homeostatic Model Assessment for Insulin Resistance; LDL: low density lipoprotein; HDL: high density lipoprotein; CRP: high-sensitive C-reactive protein; IL-6: interleukin-6; TNF-α: tumor necrosis factor alpha; IMTc: carotid artery intima-media thickness.

Independent variable: Control= 0 (reference); HIV-infected not treated= 1; HIV-infected non PI-ART= 2; HIV-infected PI-ART= 3.

† ln and †† square root transformation. * Significant at least <0.05; ** p-value of HIV-infection. All models were adjusted by age, sex and pubertal status.
