# Supplementary material for: Dyslipidemia, chronic inflammation, and subclinical atherosclerosis in children and adolescents infected with HIV: The PositHIVe Health Study
Source: PLoS One. 2018 Jan 10;13(1):e0190785. doi: 10.1371/journal.pone.0190785 (PMC5761890; doi:10.1371/journal.pone.0190785)
Supplement: S2 Table — Legend: * ln transformation; † square root transformation. a Adjusted for age, sex, maturity, body max index and use of protease inhibitors in antiretroviral treatment. CRP: C-reactive protein; IL-6: interleukin-6; TNF-α: tumor necrosis factor alpha. (DOCX) [file pone.0190785.s002.docx]

|  | **HIV viral load**  **≤ 40 copies.mL**  **(n = 44)** | **HIV viral load**  **> 40 copies.mL**  **(n = 21)** |  |  | **HIV viral load**  **≤ 40 copies.mL**  **(n = 44)** | **HIV viral load**  **> 40 copies.mL**  **(n = 21)** |  |  |
| --- | --- | --- | --- | --- | --- | --- | --- | --- |
|  | **Crude Analysis** | | |  | **Adjusted Analysis ^a^** | | | |
|  | **Mean (Standard Error)** | | **F** | ***p*** | **Mean (Standard Error)** | | **F** | ***p*** |
|  |  |  |  |  |  |  |  |  |
| CRP (mg.L^-1^)* | 3.2 (0.638) | 2.9 (0.923) | 1.656 | 0.203 | 3.4 (0.645) | 2.6 (0.952) | 1.371 | 0.246 |
| IL-6 (pg.mL^-1^)† | 2.79 (0.691) | 2.11 (1.000) | 0.017 | 0.897 | 2.98 (0.712) | 1.71 (1.052) | 0.152 | 0.698 |
| TNF-α (pg.mL^-1^)† | 1.08 (0.331) | 0.80 (0.479) | 0.006 | 0.938 | 1.155 (0.338) | 0.648 (0.500) | 0.191 | 0.664 |

**S2 Table. Crude and adjusted comparison of inflammatory markers between patients had achieved undetectable viral load (HIV RNA ≤ 40 copies.ml) and patients with > 40 copies/mL.**

* ln transformation; † square root transformation. ^a^ Adjusted for age, sex, maturity, body max index and use of protease inhibitors in antiretroviral treatment

CRP: C-reactive protein; IL-6: interleukin-6; TNF-α: tumor necrosis factor alpha.
